# Testis transcriptome profiling identified genes involved in spermatogenic arrest of cattleyak

**DOI:** 10.1371/journal.pone.0229503

**Published:** 2020-02-24

**Authors:** Shixin Wu, TserangDonko Mipam, Chuanfei Xu, Wangsheng Zhao, Mujahid Ali Shah, Chuanping Yi, Hui Luo, Xin Cai, Jincheng Zhong

**Affiliations:** 1 Key Laboratory of Qinghai-Tibetan Plateau Animal Genetic Resource Reservation and Utilization, Sichuan Province and Ministry of Education, Southwest Minzu University, Chengdu, China; 2 School of Life Science and Engineering, Southwest University of Science and Technology, Mianyang, Sichuan, China; China Agricultural University, CHINA

## Abstract

**Background:**

Cattleyak are the hybrid offspring between cattle and yak and combine yak hardiness with cattle productivity. Much attempt has been made to examine the mechanisms of male sterility caused by spermatogenic arrest, but yet there is no research systematically and precisely elucidated testis gene expression profiling between cattleyak and yak.

**Methods:**

To explore the higher resolution comparative transcriptome map between the testes of yak and cattleyak, and further analyze the mRNA expression dynamics of spermatogenic arrest in cattleyak. We characterized the comparative transcriptome profile from the testes of yak and cattleyak using high-throughput sequencing. Then we used quantitative analysis to validate several differentially expressed genes (DEGs) in testicular tissue and spermatogenic cells.

**Results:**

Testis transcriptome profiling identified 6477 DEGs (2919 upregulated and 3558 downregulated) between cattleyak and yak. Further analysis revealed that the marker genes and apoptosis regulatory genes for undifferentiated spermatogonia were upregulated, while the genes for differentiation maintenance were downregulated in cattleyak. A majority of DEGs associated with mitotic checkpoint, and cell cycle progression were downregulated in cattleyak during spermatogonial mitosis. Furthermore, almost all DEGs related to synaptonemal complex assembly, and meiotic progression presented no sign of expression in cattleyak. Even worse, dozens of genes involved in acrosome formation, and flagellar development were dominantly downregulated in cattleyak.

**Conclusion:**

DEGs indicated that spermatogenic arrest of cattleyak may originate from the differentiation stage of spermatogonial stem cells and be aggravated during spermatogonial mitosis and spermatocyte meiosis, which contributes to the scarcely presented sperms in cattleyak.

## Introduction

Cattleyaks are the hybrid offspring between cattle & yak, and exhibit the same adaptability as yak to harsh environments which are characteristic of high elevation, oxygen deficiency and low temperatures, and simultaneously, the hybrids possess higher capability than yak in terms of productive potential. Actually, cattleyaks have contributed much more to animal husbandry development of the areas surrounding Qinghai-Tibet plateau in the long history [1]. Obviously, cattleyak reproduction is the key step in yak hybrid breeding practice. However, male sterility of cattleyak prevents fixation the excellent gene combinations in their F1 generations [2]. Therefore, investigation of the mechanisms of male sterility of cattleyak has both theoretical and practical significance in yak breeding. Many attempts have been made to examine the mechanisms of male sterility of cattleyak caused by spermatogenic arrest, but so far there is no research systematically and precisely elucidated the mechanisms. Some research has been performed to compare the anatomical and histological structures of testis between cattleyak and yak, and the karyotype of spermatogenic cells as well. Testicular histology indicated that the walls of seminiferous tubules of cattleyak were significantly thinner and the number of spermatogenic cells degraded at the stage from spermatogonia, primary and secondary spermatocytes [3]. Chromosome numbers (2n = 60) in primary spermatocytes of cattleyak were found to be identical to those of cattle and yak [4], morphological abnormalities of autosomal synaptonemal complex were observed to be common in most primary spermatocytes of cattleyak and no synaptonemal XY chromosomes were observed in cattleyak [4].

During the past few years, continuously increased works were conducted to investigate the mechanisms of spermatogenic arrest of cattleyak by comparative studies of the gene expression associated with meiosis on molecular level. The lower expression of *SYCP3* and *Bvh* resulted from higher methylation in testis of cattleyak were presumed to be related to meiotic arrest during spermatogenesis in cattleyak [5, 6]. Besides, the downregulation of *Dmc1*, *DDX4* and *Dmrt7* involved in meiotic recombination, male sterility and sexual development were also considered to be associated with spermatogenic arrest in cattleyak [1, 2, 7]. In previous study, we compared the testis gene expression profiles between cattleyak and yak by RNA-seq and identified 2960 genes differentially expressed, in which several downregulated genes in cattleyak were associated with cell cycle progression, meiosis and sperm components [8]. However, we only obtained the single-end reads averaged 2.2 GB and all these data was far less to analyze testis gene expression profiles between cattleyak and yak [8].

Spermatogenesis is partially regulated through the action of genes and the functions of most those have not been determined. Recent advances in the new generation high-throughput sequencing have enabled in-depth analyses of spermatogenesis-related genes, which will contribute to the understanding of the roles played by genes in spermatogenic arrest of cattleyak. Therefore, we explored gene expression profiles between the testis of cattleyak and yak by deep sequencing and analyzing 150-bp paired-end reads with the aims to bring much more insight into mechanisms for spermatogenic arrest of cattleyak.

## Materials and methods

### Sample collection

Three male cattleyaks (CY1, CY2 and CY3) and three Maiwa yaks (YK1, YK2 and YK3) were selected from a pasture in Hongyuan county, Sichuan province of China. Here, all the cattleyaks were F1 generations from the crossbred offspring between Simmental cattle (♂) and Maiwa yak (♀). All the animals were 12 months old and the testis sample of each animal was obtained by veterinary surgical operation. After removing the epididymis, fat and fascia tissues from each testis, three slices of testicular samples were crosscut from the middle of testis by fine scale dissection. One crosscut slice was fixed with 4% paraformaldehyde for histological observation, and others were immediately immersed into liquid nitrogen and stored until total RNA extraction. The experimental animal procedures were followed in accordance with the approved protocols of Sichuan Province, PR China for the Biological Studies Animal Care and Use Committee. And all protocols were approved by the Institutional Review Board of Southwest Minzu University and Southwest University of Science and Technology.

### Histological analysis of testis samples

The testicular samples were processed routinely by paraffin embedding, and hematoxylin & eosin staining method was applied to observe the histological structures of the micro-sections from testicular samples of cattleyak and yak. Micrographs for each sample were observed through BA400Digital camera system and cropped by using Motic Images Advanced (Motic Electric Group Co., Ltd, Xiamen, China). All the images for testicular sample were magnified 400 times for further analysis.

### RNA isolation and cDNA library construction

Total RNA for each sample was isolated by using TRIzol reagent. The concentration, purity and integrity were assessed to meet the requirement of the Illumina HiSeqTM 2500 sequencing. Equal amounts of total RNA were pooled for constructing cDNA libraries. These libraries were constructed using the TruSeq RNA Sample Preparation Kit (Illumina, San Diego, CA) according to the manufacturer’s instructions after removal of ribosomal RNA. The qualification and quantification of sample libraries were checked on 2100 Bioanalyzer and Qubit 2.0, respectively.

### Transcriptome sequencing and gene expression analysis

The library of each sample was sequenced at the Shanghai Biotechnology Corporation (Shanghai, China) on Illumina HiSeqTM 2500 platform. The sequence reads were demultiplexed using the CASAVA v1.8.2 software (Illumina, San Diego, CA) and the quality inspection was based on the FastQC algorithm (http://www.bioinformatics.babraham.ac.uk/projects/fastqc/). Clean data were obtained after filtering out reads with the sequence of adapter, low quality and length less than 25nt reads from raw data through Seqtk v1.2-r94 (https://github.com/lh3/seqtk). After quality control, the 150-bp paired-end clean reads were aligned to the UMD3.1.75 *Bos taurus* reference genome [9, 10] (ftp://ftp.ensembl.org/pub/release-85/fasta/bostaurus/dna/Bostaurus.UMD3.1.dna.toplevel.fa.gz) using TopHat v2.0.9 [11]. No more than 2 mismatches and 2 multihits were allowed in the mapping genome using SAMtools v1.3 and Linux commands [12]. The paired reads mapped uniquely and properly were then assembled with Cufflinks v2.1.1 [13] using Ensembl’s bovine gene annotation. All assembled transcripts were then merged using the cuffcompare v2.2.1 [14]. The analyses of randomness, relative position and distribution of reads, and coverage of genes were same as that described in our previous works [8].

The expression levels for protein-coding genes were estimated by using standardized FPKM method [15]. HTSeq v0.5.4 [16] was used to count the fragments per gene after being aligned using TopHat v2.0.9, and Trimmed Mean of M Values [17] was used for normalization. Cluster analysis of gene expression patterns for all cattleyaks and yaks were performed by using cluster and java Treeview software. EdgeR was used for DEGs analysis [18] and false discovery rate was controlled to definite the threshold of P-value [19]. The DEGs between cattleyak and yak were screened by using p-value < 0.05 and the absolute value of Log_2_Ratio ≥ 1 as the thresholds.

### Gene ontology (GO) and pathway enrichment of DEGs

GO enrichment analysis was used to map all DEGs to GO terms in the database (http://www.geneontology.org/), calculate the numbers of DEGs for every term and compare with the whole genome background to screen significantly enriched GO terms (http://smd.stanford.edu/help/GOTermFinder/GOTermFinder_help.shtml/). KEGG (Kyoto Encyclopedia of Genes and Genomes) was used to identify significantly enriched pathways in DEGs which were presumed to be associated with spermatogenesis of cattleyak and yak. The methods used for significantly enriched GO term and pathway enrichment were all described in our previous works [8].

### Isolation and identification of spermatogenic cells from testis tissue of cattleyak and yak

The process of testicular tissue collecting from yaks (n = 3) and cattleyaks (n = 3) for cell isolation is the same as that described above. We isolated the spermatogenic cells (including spermatogonia and spermatocytes) from these two bovid species using STA-PUT velocity sedimentation, and the protocol was performed by referring to our previous study [20]. Firstly, the spermatogenic cells were preliminarily identified based on the morphological characteristics and size differences at different spermatogenic stages. The different type of spermatogenic cells were recycled and then stored in an appropriate amount of TRIzol reagent for RNA extraction. The total RNA of the isolated spermatogenic cells was extracted and the concentration was determined. RT-PCR was performed to identify the specific spermatogenic cells using the selected marker genes (*CD9*, *UCHL1*, *RET* and *THY1*) for spermatogonia, and the genes (*TESMIN*, *NUMB*, *SYCP1* and *PIWIL2*) for spermatocytes. Qualified RNA from testicular tissues of three yaks and three cattleyaks was detected using PrimeScript^TM^ One Step RT-PCR Kit Ver.2 (Takara), and the amplification procedure was: reverse transcription at 50°C for 30 min, pre-denaturation at 94°C for 2 min, and 30 cycles of denaturation at 94°C for 30 s, annealing at 59°C for 30 s and extension at 72°C for 20 s, respectively. The specific primers used for the identification were designed and *GAPDH* was used as internal reference (S1 Table).

### Quantitative RT-PCR validation and analysis for the RNA-seq data and spermatogenic cells

To validate the reliability of gene expression data from RNA-seq, qRT-PCR was conducted on the total RNAs isolated from testis tissues or spermatogenic cells to analyze the expression of representative genes selected from DEGs. Reverse transcription of total RNA (1 μg) was performed by using PrimeScript^TM^ RT reagent Kit (Takara) according to the manufacturer’s instructions. Real time RT-PCR was detected by using SYBR Premix Ex Taq (Tli RNaseH Plus) (Takara) in CFX96 Touch^TM^ Real-Time PCR Detection System (BIO-RAD). Each reaction volume (20 μL) comprised 5 pmoles of primers. Each PCR reaction was performed in triplicate, using the program as follows: 95°C for 30 s; 40 cycles of 95°C for 5 s and 57.5°C for 34 s, followed by 95°C for 15 s; 57.5°C for 1 min and 95°C for 15 s. The relative expression of DEGs was calculated based on the 2^-ΔΔCt^ method with *β*-*actin* gene as an endogenous reference. The primers for qRT-PCR were designed based on the gene sequence of *Bos taurus* and concluded in S1 and S2 Tables.

### Statistical analysis

Each experiment was performed in triplicate and the corresponding values obtained were presented as mean ± SEM (standard error). ANOVA (One-way analysis of variance) was employed to analyze the homogeneity of variances via Students' t-test by using GraphPad Prism7.01. The level of significance was presented as *P< 0.05, **P< 0.01 or ***P< 0.001.

## Results

### Histological characteristics of testis for cattleyak and yak

**Fig 1:**
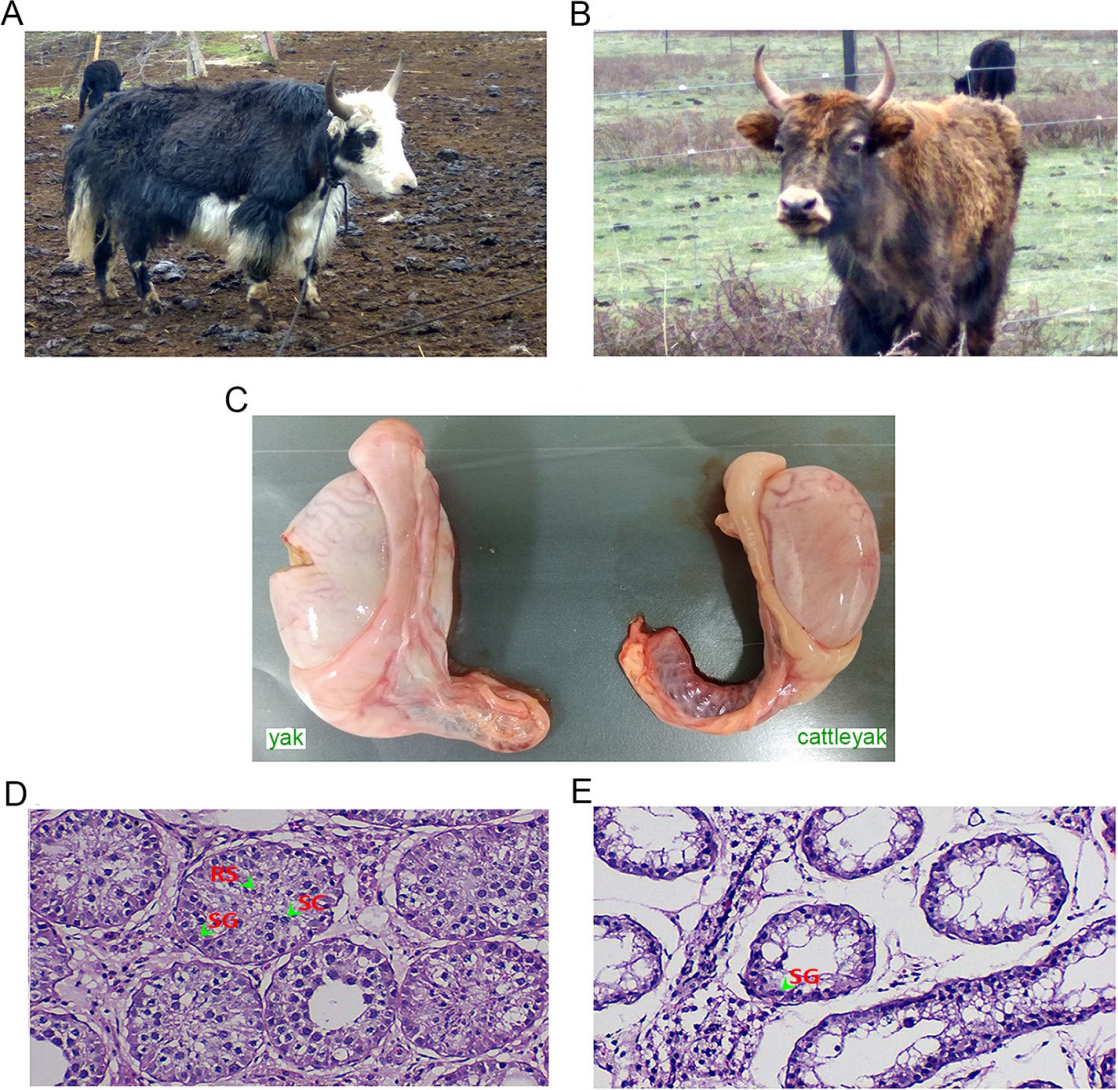
Morphological and histological differences between the testis of cattleyak and yak. (A) Yak. (B) Cattleyak. (C) The testis of yak is significantly larger than that of cattleyak with the same age of yak. The divergences between the testis histology of yak (D) and cattleyak (E). The spermatogenic cells were indicated by arrowhead. SG, SC and RS denote spermatogonium, spermatocyte and round spermatid, respectively.

As the hybrid of cattle and yak (Fig 1A), cattleyak (Fig 1B) exhibits remarkable heterosis between cattle and yak, which also inherits the excellent traits of adaptability and performance from both cattle and yak. The male sterility of cattleyak may be partially resulted from their poor development of testis, as the testes sampled from cattleyak were significantly smaller and lighter (cattleyak: 20.670±2.201 g versus yak: 30.947±1.581 g, P <0.01) than those from yak (Fig 1C, S3 Table). The seminiferous tubule of yak (Fig 1D) showed normal characteristics and was abundant in all types of germ cells (spermatogonia, spermatocytes and spermatids) in differentiation throughout from the basement membrane to the lumen, while the inner components was much thinner under the basement membrane of seminiferous tubule in cattleyak (Fig 1E) and spermatogonia were the main type of germ cells.

### RNA sequencing and mapping of reads to the reference genome

To identify the genes involved in spermatogenic arrest of cattleyak, we sequenced and compared the testis transcriptome of cattleyak and yak. An average of 10.75 GB of pair-end reads were obtained for the six samples, ranging from 9.7 to 13.2 GB (NCBI accession numbers: PRJNA509997, PRJNA510216, PRJNA510224, PRJNA510232, PRJNA510475 and PRJNA510552). The raw reads produced by sequencing ranged from 64642848 to 87657186 among the six samples (S4 Table). The clean reads ranged from 55495355 to 75311337, with the clean ratio ranging from 85.85% to 88.19%. The trimmed rRNAs ranged from 51034654 to 69110087, with the rRNA ratio ranging from 5.9% to 11.6%. The total mapped reads to the reference genome ranged from 37088324 to 56081965, with the rate ranging from 72.67% to 81.15%, in which the mapped unique reads ranged from 36301478 to 55326377 (Table 1).

**Table 1:** Sequencing statistics summary of samples analyzed in this study.

Samples ID	All reads	Mapped reads	Mapped Pair Reads	Mapped broken-pair reads	Mapped Unique reads	Mapped Multi reads	Mapping ratio
CY1	69110087	56081965	51423578	4658387	55326377	755588	81.15%
CY2	55664331	43291694	39086926	4204768	42428170	863524	77.77%
CY3	59655152	46004648	41250838	4753810	45110000	894648	77.12%
YK1	51034654	37088324	32221366	4866958	36301478	786846	72.67%
YK2	52534929	39074577	33785800	5288777	38294379	780198	74.38%
YK3	53364628	39112296	33598202	5514094	38409628	702668	73.29%

Mapping ratio = Mapped reads/All reads, Mapped Unique reads = reads that have only one match site in the genome.

**Fig 2:**
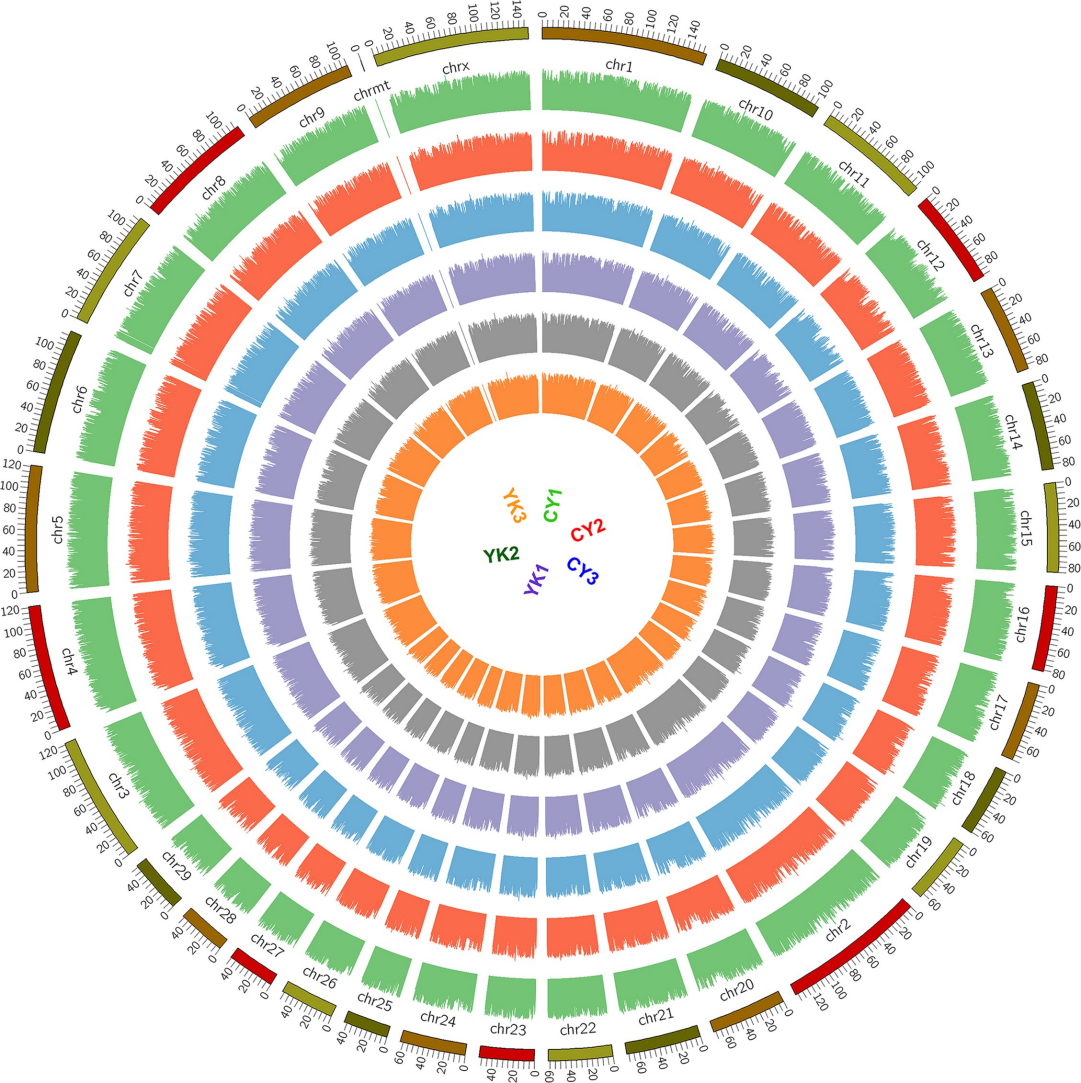
Genome coverage distribution of reads on chromosome. The coverage distribution of reads from six samples was shown in a window of 1K in cattle genome. The most outer ring donates genome and each inner ring donates the chromosome coverage for each sample.

As the reads from six samples in this work were originated from total RNAs trimmed off rRNAs, the ratios of reads mapped to the region of gene were higher than those to the intergenic region (S1 Fig). Among the gene regions, approximately equal amount of reads were mapped to the coding and intron regions. In contrast, lower amount of reads were mapped to noncoding regions and the lowest ratio of reads were mapped to splicing regions. Cattleyak and yak exhibited the similar genome coverage pattern of reads distributed on each chromosome (Fig 2). Overall, the genome coverage of reads on ChrX, Chr1, Chr2, Chr3, Chr4 and Chr5 was much higher than those distributed on Chr23, Chr25, Chr26, Chr27, Chr28 and Chr29, which was in accordance with the length and gene volume of these chromosomes.

The analysis of global Pearson correlation coefficients indicated a distinct gene expression pattern among the six testis samples (S2 Fig). The average correlation coefficients between the replicates within each group were as high as 0.95, while those between the individuals in different group were only 0.87. Systematic clustering also indicated that three replicates of cattleyak (CY1, CY2, and CY3) were clustered in a group and three yaks (YK1, YK2, and YK3) were grouped together (S3 Fig). Therefore, the biological replicates for each group exhibited higher similarity, while variability of the gene expression was observed between the two groups.

### Differentially expressed genes between the testis transcriptomes of cattleyak and yak

**Fig 3:**
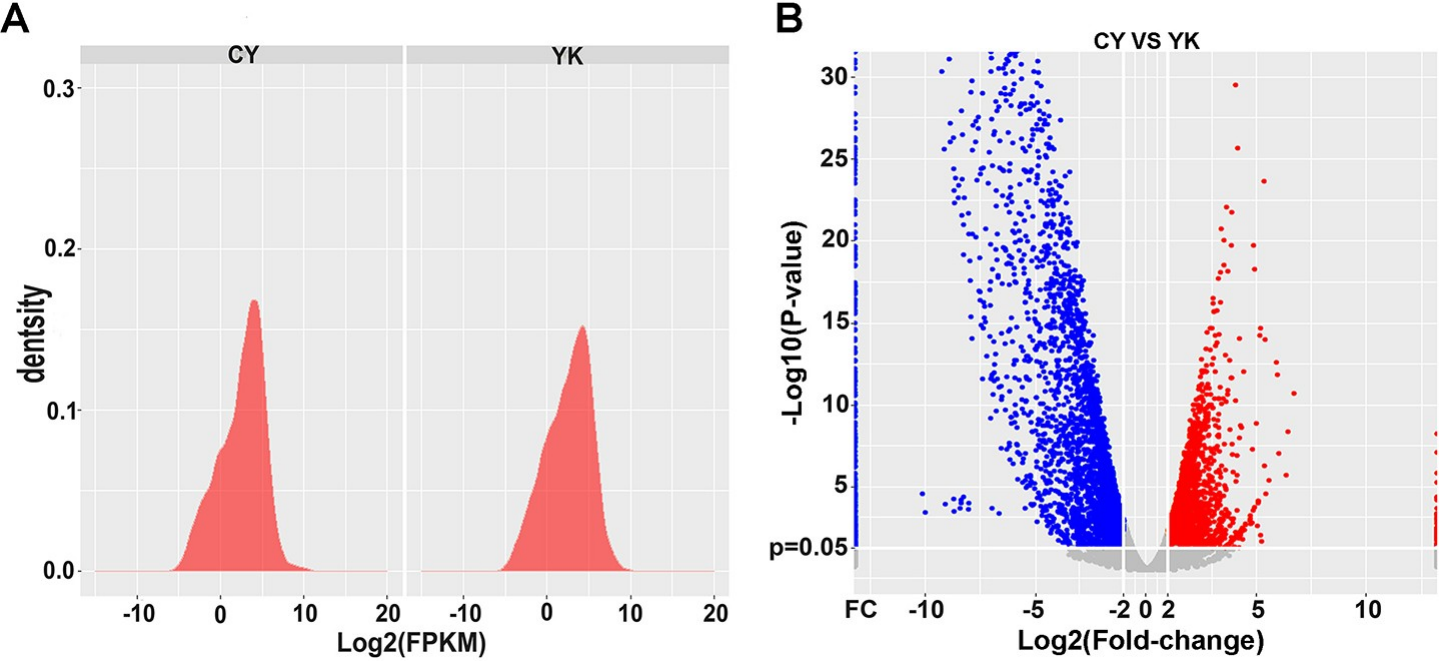
Gene expression, plots and enrichments of differentially expressed genes. (A) Gene expression distribution of cattleyak and yak. (B) Volcano plot of differentially expressed genes. Plots were constructed using log_**2**_ of fold-changes (CY/YK) and -log_**10**_ of p values. The vertical lines indicate differentially expressed genes with the log_**2**_ of fold-changes (CY/YK) ≥2 or ≤2, and the horizontal lines indicate differentially expressed genes with the -log_**10**_ of p values ≥0.05. The red points represent the upregulated genes and the blue ones represent the downregulated genes.

**Fig 4:**
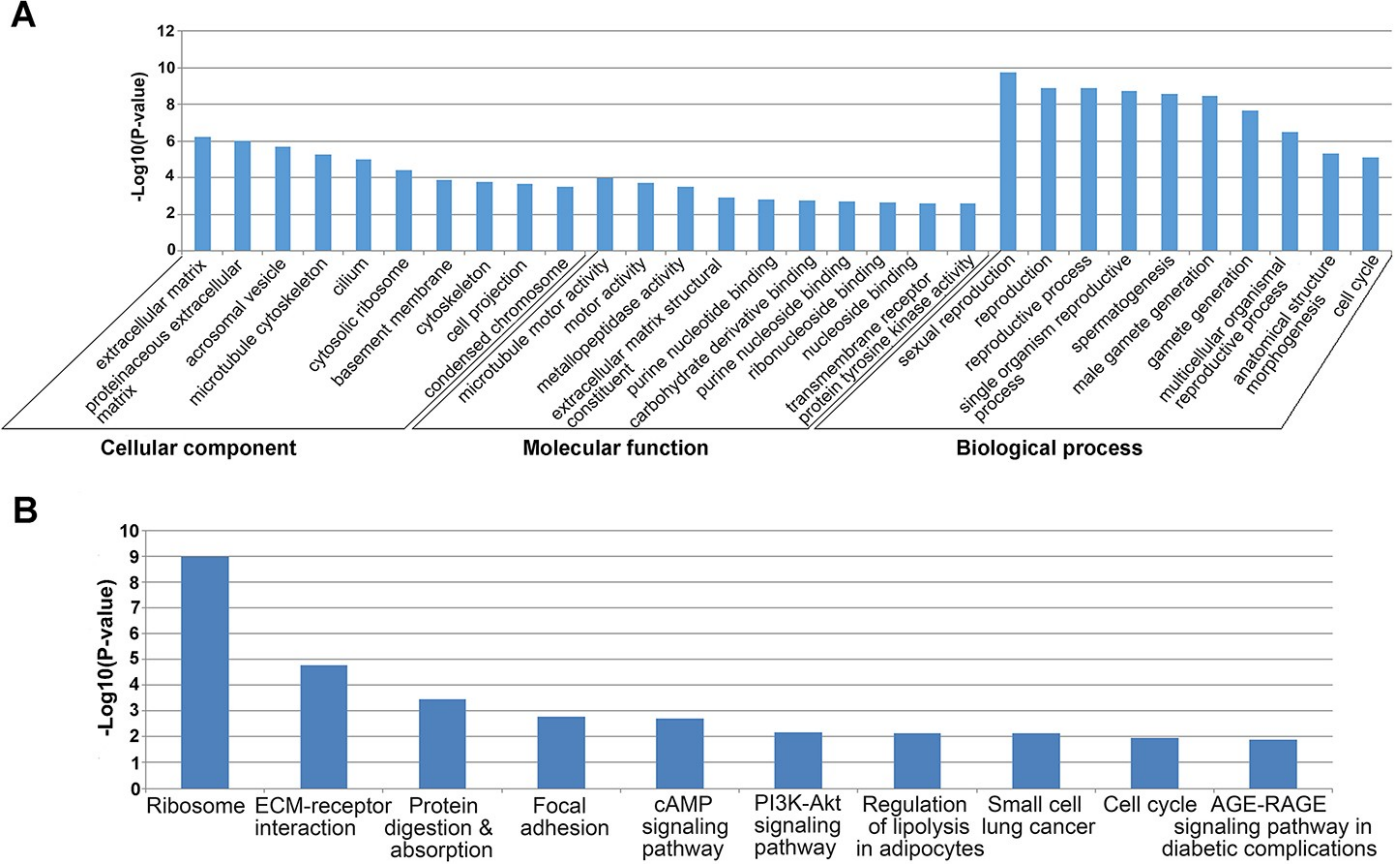
Enrichments of differentially expressed genes. (A) The top 10 items of GO enrichments of DEGs for the category of cellular component, biological process and molecular function, respectively. GO terms in each ontological category were ranked according to decreased -log_**10**_ of p values listed on the y-axis. (B) The top 10 pathways of KEGG enrichments of DEGs were ranked according to decreased -log_**10**_ of p values listed on the y-axis.

The gene expression density between cattleyak and yak exhibited the similar patterns (Fig 3A), while the number and range (-Log_10_p-value) of downregulated genes in cattleyak was significantly larger than those of upregulated genes (Fig 3B). In total, 6477 genes were identified to be DEGs, in which 2919 were upregulated and 3558 were downregulated in cattleyak (S5 Table). All DEGs between cattleyak and yak were subjected to GO enrichment based on their cellular component, molecular function, and biological process. The top listed 10 items for each GO category was ranked according to the decreased -log_10_ of p values (Fig 4A), in which the most significantly enriched three terms involved in biological process were sexual reproduction (p = 1.92E-10), reproduction (p = 1.25E-09) and reproductive process (p = 1.35E-09). Extracellular matrix (p = 5.81E-07) and microtubule motor activity (p = 9.97E-05) were the most significantly enriched term involved in cellular component and molecular function, respectively (Fig 4A, S6 Table). The significantly enriched GO items of DEGs were further summarized in the specific spermatogenic processes and listed in a descending order of p-values within each specific process (S6 Table).

Spermatogenesis initiates from spermatogonial stem cells (SSCs) which are a subset of undifferentiated spermatogonia including A_s_ or some A_pr_ and A_al_ spermatogonia. The marker genes for SSCs (*PAX7*, *ZBTB16* and *RET*) and the genes for SSCs self-renewal (*GDNF* and *MYC*) were upregulated in cattleyak, while the gene for SSCs maintenance (*UTF1*) were downregulated in cattleyak (Table 2). Furthermore, the genes involved in SSCs apoptosis (*NOTCH1*, *LHX1* and *BCL6B*) were also differentially expressed between cattleyak and yak.

**Table 2:** Differentially expressed genes associated with undifferentiated spermatogonia between cattleyaks (CY) and yaks (YK).

Gene name	Description	log_2_FC (CY/YK)	P value	Up/down
Marker genes of undifferentiated spermatogonia				
PAX7	paired box 7	2.7884	0.0019	UP
ZBTB16 (PLZF)	zinc finger and BTB domain containing 16	1.4655	4.03E-05	UP
RET	proto-oncogene tyrosine-protein kinase receptor Ret precursor	1.9442	7.81E-05	UP
Genes associated with the self-renewal of spermatogonial stem cells				
GDNF	glial cell derived neurotrophic factor	3.7732	6.48E-05	UP
MYC	v-myc avian myelocytomatosis viral oncogene homolog	2.1814	3.00E-05	UP
Genes associated with the maintenance of spermatogonia stem cells				
UTF1	undifferentiated embryonic cell transcription factor 1	-1.5375	0.0139	DOWN
Genes associated with the apoptosis of spermatogonia stem cells				
NOTCH1	notch 1	1.2643	0.0027	UP
LHX1	LIM homeobox 1	-2.0923	3.62E-04	DOWN
BCL6B	B-cell CLL/lymphoma 6B	-1.7877	3.46E-04	DOWN

Mitotic proliferation of spermatogonia is the first phase comprised in spermatogenesis, during which a majority of DEGs associated with genome stability (*CCDC113*, *RAD18*, *DNA2* and *FANCD2*), DNA replication (*CLSPN* and *TICRR*), mitotic checkpoint (*CDC6*, *SPDL1*, *BUB1*, *CDK1*, *CHEK1*, *MAD2L2*, *STIL*, *NEK11*, *SPC25*, *TRIP13* and *SKA3*), centrosome protein composition (*CCDC38*, *CCDC81*, *CCDC146*, *C4orf47*, *C7orf31*, *CEP41*, *CEP55*,*CEP63*, *CEP72*, *CEP85*, *CEP128*, *CEP152*, *CEP164*, *CEP192*, *CEP350*, *CENPF*, *CENPJ* and *CNTRL*), spindle dynamics (*KIF2A*, *KIF2B*, *KIF2C*, *KIF3B*, *KIF14*, *KIF9*, *KIF11*, *KIF18A*, *KIF18B*, *KIF22*, *KIF23* and *KIF*) and cell cycle progression (*PIWIL2*, *TP53*, *MYBL2*, *E2F7*, *FOXM1*, *CDCA3*, *CDC20* and *UBE2C*) were downregulated in cattleyak. During the period of meiosis, almost all genes related to synaptonemal complex assembly (*SYCP1*, *SYCP2*, *SYCE1*, *SYCE1L* and *SYCE3*), meiotic recombination (*TEX11*, *TEX12*, *MEIOB*, *HFM1*, *CNTD1*, *CCNB1IP1* and *STRA13*) and meiotic progression (*MSH4*, *MEI1*, *CCDC155*, *STAG3*, *SMC1B*, *REC8*, *MAEL*, *HORMAD1*, *SPATA22*, *BOLL*, *MNS1*, *M1AP* and *SGO2*) were not expressed in cattleyak. The process of spermatogenesis also includes subsequent cell part morphogenesis and sperm formation, and dozens of genes involved in acrosome formation (*SPACA1*, *SPACA4*, *SPACA8*, *SPACA9*, *TBC1D21*, *ATP8B3*, *ACR*, *ACRBP*, *ACRV1*, *CAPZA3*, *TXNDC8* and *DPY19L2*), ciliogenesis (*KIF19*, *KIF24*, *KIF27*, *IFT57*, *IFT81*, *IFT140*, *RPGRIP1L*, *DCDC2*, *CCT2*, *CCT3*, *CCT5* and *CCT7*) and flagellar development (*LRGUK*, *SPEF1*, *SPEF2*, *DNAH1*, *DNAH7*, *DNAH8*, *DNAI2*, *PACRG*, *ODF3*, *TEKT1*, *TEKT2* and *TEKT4*) were dominantly downregulated in cattleyak. All these further contributed to the drastically decreased expression of genes associated with sperm motility (*CATSPER1*, *CATSPER2*, *CATSPER3*, *AKAP3*, *AKAP4*, *SEPT12*, *SORD*, *SLC9C1*, *LDHC* and *ATP1A4*), acrosome reaction (*FAM170B*, *IQCF1*, *TRIM36*, *CABYR*, *PCSK4* and *GLRA1*) and fertilization (*STK31*, *PAFAH1B1*, *INSL6*, *ZPBP*, *WBP2NL*, *PLCZ1* and *GLRA1*) in cattleyak (S6 Table).

On the other hand, a larger amount of genes involved in such biological processes as collagen fibers formation/microfibril assembly (*ADAMTS1*, *ADAMTS3*, *ADAMTS12*, *ADAMTS14*, *ADAMTS15*, *COL1A1*, *COL1A2*, *COL5A1*, *COL5A2*, *COL6A1*, *COL6A2*, *COL6A3*, *COL14A1*, *COL27A1*, *MFAP2*, *MFAP4* and *MFAP5*), cell adhesion (*LAMA1*, *LAMA2*, *LAMB1*, *LAMB2*, *LAMB3*, *LAMC1*, *TGFBI*, *TGFBP7*, *NPNT*, *FREM2*, *SPON1*, *CD151*, *FBLN1*, *GLG1*, *LGALS3BP*, *MYOC*, *ADAMTS6*, *THBS2*, *EFEMP1*, *FRAS1* and *ITGA10*) and extracellular matrix interaction (*ADAMTSL2*, *COL4A1*, *COL17A1*, *SOD3*, *SPARC*, *DAG1*, *LAMA3*, *LAMC3*, *CASK*, *SSC5D*, *NID1*, *PRELP*, *VCAN*, *COMP*, *SMAD3* and *ITGA11*) were dominantly upregulated in cattleyak with respect to yak (S6 Table).

All DEGs between cattleyak and yak were mapped to the reference pathways in KEGG database. In total, 32 significantly enriched pathways were obtained (Fig 4B, S7 Table) (P<0.05), in which the top-listed three ones were ribosome (p = 1.09E-09), ECM-receptor interaction (p = 1.63E-05) and protein digestion and absorption (p = 0.000358). Further analysis of the top listed pathways indicated that dozens of genes encoding ribosomal proteins (*RPL7A*, *RPL12*, *RPL13*, *RPL19*, *RPL26*, *RPL29*, *RPL32*, *RPL37*, *RPS6*, *RPS12*, *RPS15*, *RPS18*, *RPS20*, *RPS27A*, *RPLP1*, *RPS5*, *RPS27*, *RPS29*, etc.) and extracellular matrix assembly and interaction (*ITGA3*, *ITGA4*, *ITGA6*, *ITGA10*, *ITGAV*, *LAMA2*, *LAMB1*, *LAMB2*, *LAMC1*, *COL1A1*, *COL4A1*, *COL4A5*, *COL9A2*, *TNC*, *DAG1*, *CHAD*, *SV2A*, etc.) were upregulated in cattleyak with respect to yak (S7 Table).

### Identification of spermatogenic cells from yak and cattleyak

**Fig 5:**
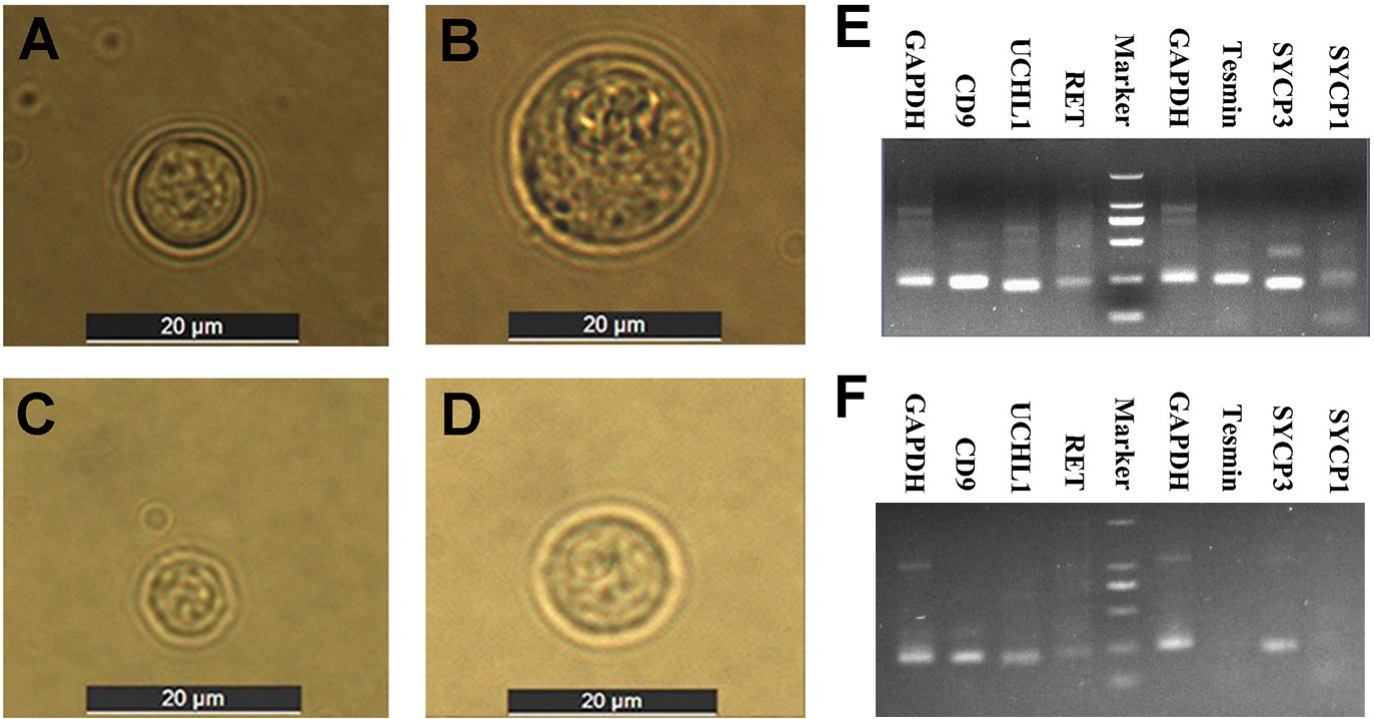
Isolation and identification of spermatogonia and spermatocytes. The images of spermatogonia (A, C) and spermatocytes (B, D) from yaks and cattleyaks under the inverted microscope with scale bars = 20 μm. The expression of marker genes for the spermatogonia and spermatocytes isolated from yaks (E) and cattleyaks (F).

Spermatogenic cells comprised spermatogonia and spermatocytes were isolated from yak and cattleyak using STA-PUT velocity sedimentation [20]. For the yak, the percentage of spermatogonia (Fig 5A) and spermatocytes (Fig 5B) was 75.36% and 24.64%, respectively; while the percentage of spermatogonia (Fig 5C) and spermatocytes (Fig 5D) for the cattleyak was 79.71% and 20.29%, respectively (S4 Fig, S8 Table). Subsequent detection of marker gene expressions further confirmed the components of the isolated spermatogenic cells. RT-PCR analysis showed that *CD9*, *UCHL1* and *RET* were expressed in spermatogonia while *Tesmin* and *SYCP3* were expressed in spermatocytes from yak (Fig 5E). In contrast, *CD9*, *UCHL1* and *RET* were also detected in spermatogonia while only *SYCP3* was detected in spermatocytes from cattleyak (Fig 5F).

### Validation of representative differentially expressed genes from testis and spermatogenic cells

To validate the identified DEGs between cattleyak and yak from RNA-seq, we randomly selected 19 DEGs (*CCDC113*, *CHEK1*, *CEP128*, *KIF18B*, *PIWIL2*, *CDCA3*, *TEX12*, *MEIOB*, *STAG3*, *SPACA1*, *CCT2*, *SPEF1*, *TEKT1*, *CATSPER1*, *ZPBP*, *ADAMTS1*, *COL1A1*, *LAMA1* and *DAG1*) involved in the process of spermatogenesis and examined their expression levels by qRT-PCR (S2 Table). Similar to what observed in RNA-seq, the expression of the genes involved in mitotic proliferation of spermatogonia, meiosis, spermiogenesis, sperm motility fertilization were downregulated in cattleyak with respect to yak, whereas those associated with extracellular matrix composition and interaction were upregulated in cattleyak (S5 Fig). In addition, in order to verify whether the DEGs are consistent in spermatogenic cells and testicular tissues of yak and cattleyak, several marker genes (*CDH1*, *Epcam*, *Lrp4* and *Stra8*) of spermatogenic cells (including spermatogonia and spermatocytes) and cell cycle-related genes (*CCNA1*, *CCNA2*, *CCNB1*, *CCNB2*, *CCNE1* and *CCNE2*) were detected in the spermatogenic cells isolated from the testis of yak and cattleyak. Corresponding to the sequencing results, qRT-PCR analysis showed that the expression level of all these selected genes in cattleyak were lower than that of yak (S6 Fig). Therefore, the overall expression trends of these genes were basically consisted with that of RNA-seq data, suggesting that the results of the RNA-seq data were reliable.

## Discussion

Male sterility of cattleyak caused by spermatogenic arrest greatly restricted their utilization in yak breeding. In this work, deep RNA-seq analysis of testis transcriptome between cattleyak and yak obtained paired-end reads averaged 10.75 GB, which was four times larger than the single-end reads (2.2 GB) we obtained from previous study [8]. The number of DEGs identified from these data was 6477, which was more than two times of what identified from previous study (2960 DEGs) [8]. Therefore, testis transcriptomic profiling by deep RNA-seq in this work identified much more genes involved in the whole process of spermatogenesis compared with those identified in our previous work [8], which provided much more information and brought much more insights into mechanisms for spermatogenic arrest of cattleyak.

Spermatogenesis initiates from SSCs which undergo the process of self-renewal and differentiation and then proceed to spermatogonial mitosis, spermatocyte meiosis and spermiogenesis. SSCs are a subset of undifferentiated spermatogonia which classically comprise only A_s_ spermatogonia, but A_pr_ and the shorter A_al_ spermatogonia also appear to potential SSCs [21]. *PAX7* is specific to type A_s_ spermatogonia [22] and *ZBTB16* (*PLZF*) is expressed in all undifferentiated A-spermatogonia at all stages [23]. *RET* expression coincides with *PLZF* with the progression of spermatogenesis [24]. The upregulation of these marker genes indicated the accumulation of undifferentiated spermatogonia in cattleyak. Over-production of *GDNF* from Sertoli cells leads to an over-growth and accumulation of SSCs, causing an arrest in early spermatogenesis [25]. *BCL6B*-depleted Thy1^+^ spermatogonial cells exhibited a ~2-fold increase in the frequency of apoptotic cells, providing evidence that *BCL6B* promotes both SSC self-renewal and survival [26]. In this work, upregulation of *GDNF* and downregulation of *BCL6B* and *LHX1* may lead to the accumulation and apoptosis of undifferentiated spermatogonia in cattleyak. Over expression of *NOTCH1* in male germ cells correlated with overexpression of pro-apoptotic cell markers, *Trp53* and *Trp63*, which resulted in increased apoptosis in PLZF^+^ cells and decreased sperm number and testis weight [27]. In present study, upregulation of *NOTCH1* may also have contributed to the increased apoptosis of spermatogenic cells and decreased testis weight via upregulation of *Trp53* [log_2_FC(CY/YK) = 1.1986; p = 0.0007] in cattleyak. The expression of *UTF1* is restricted to A_s_, A_pr_, and short chains of A_al_ spermatogonia and therefore maintain their ability to differentiate into A1 spermatogonia [28]. However, downregulation of *UTF1* was likely to weaken the differentiation ability and further accumulation of undifferentiated spermatogonia in cattleyak. Therefore, the accumulation and apoptosis of undifferentiated spermatogonia may contribute to spermatogenic arrest of cattleyak.

During the process of spermatogonial mitosis, a majority of DEGs associated with genome stability, DNA replication, mitotic checkpoint, centrosome protein composition, spindle dynamics and cell cycle progression were downregulated in cattleyak in this work. *FANCD2* was documented to influence replication fork processes and genome stability in response to clustered DNA double-stranded breaks (DSBs) [29]. *TICRR* was reported to be an essential checkpoint and replication regulator and involved in the initiation of DNA replication [30]. The downregulation of *CHEK1* involved in DNA repair activation may directly affect DNA damage repair in spermatogonial cells [31]. Decreased expression of these genes may affect DNA replication and the integrity of the genome in spermatogonia of cattleyak. A series of centrosomal protein (*CEP*) genes were the active component of centrosome and played a vital role in centriole biogenesis and cell cycle progression, in which *CEP152* [32] and *CENPJ* [33] were critical to centrosome function by participating in centriole formation, duplication and elongation, respectively. And their downregulation may lead to abnormal chromosomes division. A series of kinesin family (*KIF*) genes (*KIF2A*, *KIF2B*, *KIF2C*, *KIF14*, *KIF9*, *KIF11*, *KIF18A*, *KIF18B*, *KIF22* and *KIF25*) were also downregulated in cattleyak, in which *KIF2B* and *KIF22* were crucial for spindle formation and assembly and chromosome congression during mitosis [34, 35]. Downregulation of these genes may influence the spindle dynamics, and in turn affect the arrangement and division of chromosomes for dynamic turnover of the spindle was a driving force for chromosome congression and segregation in mitosis [36]. *CDCA3* was referred to as a trigger of mitotic entry and mediate destruction of mitosis [37] and *UBE2C* to control mitosis progression as an essential factor of the anaphase-promoting complex/cyclosome (APC/C) [38]. The downregulation of these genes could block up spermatogonial mitosis and further contribute to spermatogenic arrest of cattleyak.

Meiosis begins after the G2 phase in the spermatocyte cycle and undergoes two successive nuclear divisions, which is the crucial stage to produce haploid spermatids. In this work, almost all DEGs related to synaptonemal complex assembly, meiotic recombination and meiotic progression presented no sign of expression in cattleyak. Synapsis is the key step in meiosis which mediates the alignment of homologous chromosome and the formation of synaptonemal complex. *SYCP1* and *SYCP2* were expressed for normal assembly of synaptonemal complexes and meiotic synapsis during spermatocyte development [39, 40]. *MEI1* was also required for normal meiotic chromosome synapsis and was suggested to involve in cattleyak male sterility [41, 42]. *MEIOB* was involved in propagation of synapsis and regulation of recombination events [43]. The absence or downregulation of these genes may affect the correct formation of the synaptonemal complex and further affect homologous chromosome recombination and segregation during meiosis. *STAG3* [44] and *CCDC155* [45] were found to function in maintaining cohesion between sister chromosomes in meiosis I and homologous chromosome pairing during meiotic prophase in spermatocytes, respectively. Therefore, the expression deficiency or downregulation of almost all genes related to meiosis was enough to cause meiotic arrest during spermatogenesis.

What’s worse, even more genes involved in such spermiogenesis process as cell part morphogenesis, sperm formation, acrosome formation, ciliogenesis and flagellar development were dominantly downregulated in cattleyak. Intraflagellar transport (*IFT*) genes (*IFT81* and *IFT140*) were formed a tubulin-binding module that specifically mediate transport of tubulin within the cilium and involved in ciliogenesis and cilia maintenance, respectively [46, 47]. A series of chaperonin containing TCP1 subunit (*CCT*) genes (*CCT2*, *CCT3*, *CCT5*, *CCT7*) were required for ciliogenesis regulating transports vesicles to the cilia through the assembly of BBSome [48]. The downregulation of these genes was not conducive to cilia formation in spermatid of cattleyak, which, to some extent, affects sperm motility. *LRGUK* and *PACRG* were critical to flagellar development through the involvement of the early axoneme development and maintenance of functional stability of the axonemal outer doublets [49, 50]. *TEKT1* and *TEKT2* were reported to participate in the nucleation of the flagellar axoneme of mature spermatozoa and required for flagellum stability and sperm motility through function as an ODF-affiliated molecule [51, 52]. Obviously, dominant downregulation of these genes involved in spermiogenesis process would contribute to the structural deficiency and morphological abnormalities of sperm in cattleyak. All these further contributed to the drastically decreased expression of such genes as associated with sperm motility (*CATSPER1*-*CATSPER3*, *SEPT12*) [53, 54], acrosome reaction (*SPACA1*, *SPACA4*, *SPACA8*, *SPACA9*, *CABYR*, *FAM170B* and *PCSK4*) [55–58] and fertilization (*STK31*, *PLCZ1*) [59, 60] in cattleyak. Therefore, the scarcely presented abnormal sperm of cattleyak lost the function of motility and fertilization.

On the other hand, spermatogenic cells’ detachment from seminiferous basement membrane and migration towards the tubule lumen are indispensable to spermatogenesis. A larger amount of genes involved in such biological processes as collagen fibers formation/microfibril assembly, cell adhesion and extracellular matrix interaction were dominantly upregulated in cattleyak with respect to yak. A series of collagen genes (*COL1A1*, *COL1A2*, *COL5A1*, *COL5A2*, *COL6A1*, *COL6A2*, *COL6A3*, *COL14A1* and *COL27A1*) were upregulated in cattleyak, in which *COL1A1* and *COL1A2* were suggested to play potential roles in mediating spermatogenic cell detachment and migration during spermatogenesis while *COL14A1* was proposed to interact with the fibril surface and involved in the regulation of fibrillogenesis [61, 62]. *MFAP4* was involved in intercellular interactions and contribute to the elastic fiber assembly and maintenance [63]. The upregulation of the intercellular genes may be the causes of seminiferous tubule fibrosis in cattleyak. *FBLN1* was suggested to play a role in cell adhesion and migration along protein fibers within the extracellular matrix [64]. *NID1* and *PRELP* were reported to function in cell interactions with the extracellular matrix and anchor basement membranes to the underlying connective tissue extracellular matrix [65, 66]. *NPNT* was documented to play an important role in regulating cell adhesion and differentiation [67]. Therefore, upregulation of these intercellular genes may prevent spermatogenic cells detaching from basement membrane and subsequent migrating towards the seminiferous tubule lumen, which further contributed to the accumulation and apoptosis of undifferentiated spermatogonia in cattleyak.

## Conclusion

**Fig 6:**
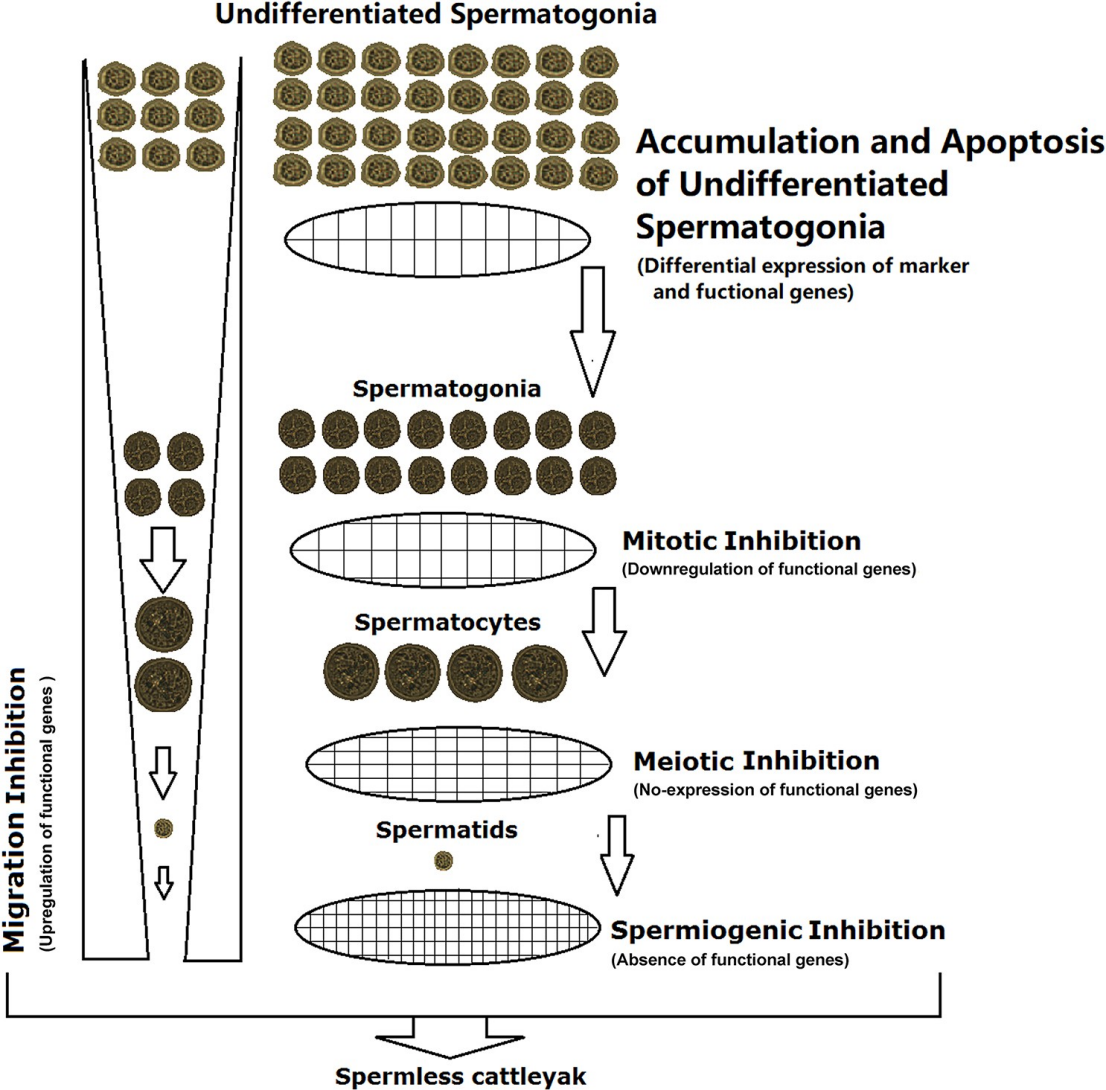
The effect of differential expression of functional genes on spermatogenesis in cattleyak with respect to yak. The downregulation, no-expression and absence of some functional genes inhibit the spermatogonial mitosis, spermatocyte meiosis and spermiogenesis, respectively; and the upregulation of others inhibit the migration of germ cells towards the seminiferous tubule lumen in cattleyak. Eventually, these differentially expressed functional genes resulted in almost no sperm in the cattleyak testis.

Compared to yak, the seminiferous tubule of cattleyak was much thinner and spermatogonia were the main type of germ cells. The effect of DEGs on the spermatogenesis of cattleyak is shown in Fig 6. On the one hand, downregulation of differentiation maintenance genes and upregulation of apoptosis regulatory genes for undifferentiated spermatogonia indicated that spermatogenic arrest of cattleyak might occur as early as the differentiation stage of SSCs and be aggravated during spermatogonial mitosis and spermatocyte meiosis, which contributes to the morphologically deficient and scarcely presented sperms lost the function of fertilization in cattleyak. On the other hand, upregulation of the genes involved in collagen fibers formation/microfibril assembly, cell adhesion and extracellular matrix interaction may impede the migration of spermatogenic cells from the basement membrane towards the seminiferous tubule lumen in cattleyak. In conclusion, our study provides novel insights into testis transcriptome profiling of cattleyak and yak, and will facilitate further experimental studies to investigate the specific functions of these molecules in spermatogenic arrest of cattleyak.

## Supporting information

S1 Table Primer sequences of the genes associated with spermatogenic cells used for RT-PCR and qRT-PCR. (DOCX)

S2 Table Primer sequences used for qRT-PCR validation of the randomly selected genes involved spermatogenesis. (DOCX)

S3 Table Statistics summary of the weight of testis sampled in this study. (DOCX)

S4 Table Sequencing statistics summary of samples analyzed in this study. (DOCX)

S5 Table Differentially expressed genes between testis transcriptome of cattleyak and yak. (XLSX)

S6 Table Summary of the significantly enriched GO items for differentially expressed genes and the list of genes involved in each item. (XLSX)

S7 Table Summary of the significantly enriched KEGG pathways for differentially expressed genes and the list of genes involved in each pathway. (XLSX)

S8 Table Statistics summary of cell composition collected by STA-PUT of yak (YK) and cattleyak (CY). (DOCX)

S1 Fig Mapping region distribution of reads in different genomic regions. The ratio of reads from six samples mapped to the gene, coding region, splicing sites, introns and non-coding regions in the genome was indicated by different colors. The coding region includes exons and the exon coding sequence and the non-coding region includes 5' UTR, 3' UTR and non-coding RNA regions.
(TIF)

S2 Fig Pearson correlation coefficient heat map of the testis transcriptomes for all samples. The darkness of the color is corresponding to the extent of correlations and the increase of Pearson correlation coefficients.
(TIF)

S3 Fig Hierarchical clustering analysis of testis transcriptomes for yak and cattleyak. CY1 (Cattleyak 1), CY2 (Cattleyak 2), CY3 (Cattleyak 3), YK1 (Yak 1), YK2 (Yak 2), YK3 (Yak 3).
(TIF)

S4 Fig Statistics of the number of testicular tissue spermatogonia and spermatocytes isolated from yak (YK) and cattleyak (CY). (TIF)

S5 Fig qRT-PCR validation of the randomly selected genes involved spermatogenesis. Data of qRT-PCR ware represented as mean±s.e.m. T-test was performed. Asterisks indicate statistical significance as compared to yak (*P < 0.05; **P < 0.01; ***P < 0.001). YK means yak, and CY means cattleyak.
(TIF)

S6 Fig qRT-PCR validation of the DEGs associated with spermatogenic cells between yaks (YK) and cattleyaks (CY). Asterisks indicate statistical significance as compared to yak (*P < 0.05; **P < 0.01; ***P < 0.001).
(TIF)

S1 Raw Image (PDF)

## List of abbreviations

RNA-seq: RNA sequencing
SSCs: spermatogonial stem cells
DEG: differentially expressed gene
GO: Gene Ontology
RT-PCR: reverse transcription polymerase chain reaction
qRT-PCR: quantitative real-time polymerase chain reaction
FPKM: fragments per kilobase of transcript per million mapped reads
KEGG: Kyoto Encyclopedia of Genes and Genomes
