# Supplementary material for: Testis transcriptome profiling identified genes involved in spermatogenic arrest of cattleyak
Source: PLoS One. 2020 Feb 24;15(2):e0229503. doi: 10.1371/journal.pone.0229503 (PMC7039509; doi:10.1371/journal.pone.0229503)
Supplement: S1 Table — (DOCX) [file pone.0229503.s001.docx]

**S1 Table. Primer sequences of the genes associated with spermatogenic cells used for RT-PCR and qRT-PCR.**

| Genes | Primers (5′-3′) | Tm (℃) | PCR product (bp) |
| --- | --- | --- | --- |
| CD9 | CD9-F: 5′-attcgactcgcagaccaaga-3′  CD9-R: 3′-tgatcacctcctccttgtgg-5′ | 58  59 | 242 |
| UCLH1 | UCLH1-F: 5′-gagttcgaggatgggtctgt-3′  UCLH1-R: 3′-ggaaaaggcatccgtccatc-5′ | 59  59 | 227 |
| RET | RET-F: 5′-agatctcacgagggatgcag-3′  RET-R: 3′-aggaccacacatcactctgg-5′ | 58  59 | 240 |
| THY1 | THY1-F: 5′-tatgagttcagcctgacccg-3′  THY1-R: 3′-ccccacacctgaccagttta-5′ | 59  59 | 247 |
| Tesmin | Tesmin-F: 5′-tgattgctttgctaacgggg-3′  Tesmin-R: 3′-ttggcctcatagcactcaca-5′ | 58  58 | 240 |
| SYCP1 | SYCP1-F: 5′-acttgtgccagatctgcaga-3′  SYCP1-R: 3′-gcagtgatacctgcttttcct-5′ | 59  58 | 247 |
| SYCP3 | SYCP3-F: 5′-cattccgggaagttggcaaa-3′  SYCP3-R: 3′-cagctccaaatctttccagca-5′ | 59  59 | 214 |
| PIWIL2 | PIWIL2-F: 5′-tccaggacgagtgcactaag-3′  PIWIL2-R: 3′-attctgtctctcactgggcc-5′ | 58  59 | 231 |
| GAPDH | GAPDH-F: 5′-catgtttgtgatgggcgtga-3′  GAPDH-R: 3′-gccagtagaagcagggatga-5′ | 58  59 | 250 |
| CDH1* | CDH1-F: 5′-cgtatcggatttggagggac-3′  CDH1-R: 3′-atcgaggaacaagagcaggg-5′ | 59  59 | 194 |
| Epcam* | Epcam-F: 5′-ggacctgagagtaaatgggg-3′  Epcam-R: 3′-tgcttttctttctggaaaca-5′ | 58  59 | 188 |
| Lrp4* | Lrp4-F: 5′-ccctgcgttcttctacca-3′  Lrp4-R: 3′-ggagtccaccgatgatgt-5′ | 59  58 | 162 |
| Stra8* | Stra8-F: 5′-gggaagacactgtgtttggc-3′  Stra8-R: 3′-cgccttttcttgcagtctcc-5′ | 58  59 | 155 |
| CCNA1* | CCNA1-F: 5′-ggtcggagaagagtacaagc-3′  CCNA1-R: 3′-aactcatctacttcgggtgg-5′ | 58  59 | 174 |
| CCNA2* | CCNA2-F: 5′-gtatttgccgtcagttatcg-3′  CCNA2-R: 3′-tattgactgttgtgcgtgct-5′ | 58  59 | 181 |
| CCNB1* | CCNB1-F: 5′-cggtgactttgcctttgtga-3′  CCNB1-R: 3′-agaaggaggaaagtgcacca-5′ | 59  59 | 229 |
| CCNB2* | CCNB2-F: 5′-tgtcagcaagcacccgaaac-3′  CCNB2-R: 3′-tctcccagtcctcggtatca-5′ | 59  58 | 179 |
| CCNE1* | CCNE1-F: 5′-aaccggataccatgaaagag-3′  CCNE1-R: 3′-tattactgtcccaaggctga-5′ | 59  58 | 162 |
| CCNE2* | CCNE2-F: 5′-acgcagtagccgtttacaagc-3′  CCNE2-R: 3′-ataatacaggcggccaacaat-5′ | 58  59 | 179 |
| β-actin* | β-actin-F: 5′-aagttctacagtgtggccga-3′  β-actin-R: 3′-gactggccccttctccttag-5′ | 59  59 | 150 |
