# Supplementary material for: Testis transcriptome profiling identified genes involved in spermatogenic arrest of cattleyak
Source: PLoS One. 2020 Feb 24;15(2):e0229503. doi: 10.1371/journal.pone.0229503 (PMC7039509; doi:10.1371/journal.pone.0229503)
Supplement: S2 Table — (DOCX) [file pone.0229503.s002.docx]

**S2 Table. Primer sequences used for qRT-PCR validation of the randomly selected genes involved spermatogenesis.**

| Genes | Primers (5′-3′) | Tm (℃) | PCR product (bp) |
| --- | --- | --- | --- |
| CCDC113 | CCDC113-F: 5′-cgaagcaggcgtaaatccaa-3′  CCDC113-R: 3′-cctcgatgatagcctcgtga-5′ | 59  59 | 172 |
| CHEK1 | CHEK1-F: 5′-cggcataataatcgggagcg-3′  CHEK1-R: 3′-ttctccagccaacattgcag-5′ | 59  59 | 150 |
| CEP128 | CEP128-F: 5′-tttaggagaggggttgagcg-3′  CEP128-R: 3′-cacttgggctgccaaatgta-5′ | 59  59 | 153 |
| KIF18B | KIF18B-F: 5′-caaagggccgaaagtctcac-3′  KIF18B-R: 3′-tcggcatacttgagggtgtt-5′ | 59  59 | 157 |
| PIWIL2 | PIWIL2-F: 5′-ggcccagtgagagacagaat-3′  PIWIL2-R: 3′-tttggggctcaggttgatct-5′ | 59  59 | 155 |
| CDCA3 | CDCA3-F: 5′-gagcaggtggagggacataa-3′  CDCA3-R: 3′-tttggggtcttcggtctcaa-5′ | 59  59 | 150 |
| TEX12 | TEX12-F: 5′-cacagctgtcctctcttgga-3′  TEX12-R: 3′-actgctgctctctcacttaaga-5′ | 59  59 | 160 |
| MEIOB | MEIOB-F: 5′-gcacttgtacaacttgcaaca-3′  MEIOB-R: 3′-tcatctaccgtgcatcccaa-5′ | 58  59 | 157 |
| STAG3 | STAG3-F: 5′-gatgctgtgaaaggtgccaa-3′  STAG3-R: 3′-acatctcaggggtcacagtg-5′ | 59  59 | 151 |
| SPACA1 | SPACA1-F: 5′-tggaagaatgtcgtggacca-3′  SPACA1-R: 3′-ataatggcttgctggtctgg-5′ | 59  58 | 151 |
| CCT2 | CCT2-F: 5′-tgcagattttgtaggcgtgg-3′  CCT2-R: 3′-cagaacaatggtgcaagcct-5′ | 59  59 | 184 |
| SPEF1 | SPEF1-F: 5′-gaagcactgcaccaactgta-3′  SPEF1-R: 3′-gagagagttggcagggacat-5′ | 58  59 | 165 |
| TEKT1 | TEKT1-F: 5′-gaatacagggagaagcgggt-3′  TEKT1-R: 3′-gattgtacttggcagagcgg-5′ | 59  59 | 163 |
| CATSPER1 | CATSPER1-F: 5′-acgttcagaagcgcaaagtt-3′  CATSPER1-R: 3′-gaaggtttcaaaggccaggg-5′ | 59  59 | 158 |
| ZPBP | ZPBP-F: 5′-accaagaagtgctcagttgc-3′  ZPBP-R: 3′-atgccactggaatgatgggt-5′ | 59  59 | 163 |
| ADAMTS1 | ADAMTS1-F: 5′-ttcttcgttttgcagcccaa-3′  ADAMTS1-R: 3′-tcctccacagatgccacatt-5′ | 59  59 | 150 |
| COL1A1 | COL1A1-F: 5′-atgtgccactctgactggaa-3′  COL1A1-R: 3′-tccttggggttcttgctgat-5′ | 59  59 | 170 |
| LAMA1 | LAMA1-F: 5′-catgcaggagaacatcacgg-3′  LAMA1-R: 3′-taacaccgccaactcttcct-5′ | 59  59 | 157 |
| DAG1 | DAG1-F: 5′-catgagcatcacagtgacgg-3′  DAG1-R: 3′-ggatgaccgtgtgcaagtac-5′ | 59  59 | 161 |
| β-actin | β-actin-F: 5′-aagttctacagtgtggccga-3′  β-actin-R: 5′-gactggccccttctccttag-3′ | 59  59 | 150 |
