# Supplementary material for: Testis transcriptome profiling identified genes involved in spermatogenic arrest of cattleyak
Source: PLoS One. 2020 Feb 24;15(2):e0229503. doi: 10.1371/journal.pone.0229503 (PMC7039509; doi:10.1371/journal.pone.0229503)
Supplement: S3 Table — (DOCX) [file pone.0229503.s003.docx]

**S3 Table. Statistics summary of the weight of testis sampled in this study.**

| Sample ID | Weight (g) | Mean±SD (g) |
| --- | --- | --- |
| CY1 | 22.439 | 20.670±2.201 |
| CY2 | 21.366 |  |
| CY3 | 18.205 |  |
| YK1 | 32.538 | 30.947±1.581 |
| YK2 | 30.928 |  |
| YK3 | 29.376 |  |
