# Supplementary material for: Testis transcriptome profiling identified genes involved in spermatogenic arrest of cattleyak
Source: PLoS One. 2020 Feb 24;15(2):e0229503. doi: 10.1371/journal.pone.0229503 (PMC7039509; doi:10.1371/journal.pone.0229503)
Supplement: S4 Table — (DOCX) [file pone.0229503.s004.docx]

**S4 Table. Sequencing statistics summary of samples analyzed in this study.**

| Sample ID | Raw reads | Clean ratio | rRNA trimed | rRNA ratio |
| --- | --- | --- | --- | --- |
| CY1 | 87657186 | 85.92% | 69110087 | 8.2% |
| CY2 | 72498478 | 86.86% | 55664331 | 11.6% |
| CY3 | 74786926 | 87.17% | 59655152 | 8.5% |
| YK1 | 64642848 | 85.85% | 51034654 | 8.0% |
| YK2 | 64679234 | 88.19% | 52534929 | 7.9% |
| YK3 | 64787060 | 87.55% | 53364628 | 5.9% |

Note：Clean ratio=(Clean reads/Raw reads)%; rRNA ratio=[(Clean reads - rRNA trimed)/ Clean reads]%
