# Supplementary material for: Testis transcriptome profiling identified genes involved in spermatogenic arrest of cattleyak
Source: PLoS One. 2020 Feb 24;15(2):e0229503. doi: 10.1371/journal.pone.0229503 (PMC7039509; doi:10.1371/journal.pone.0229503)
Supplement: S8 Table — (DOCX) [file pone.0229503.s008.docx]

**S8 Table. Statistics summary of cell composition collected by STA-PUT of yak (YK) and cattleyak (CY).**

| Bovid species  Cell types | YK1 | YK2 | YK3 | CY1 | CY2 | CY3 |
| --- | --- | --- | --- | --- | --- | --- |
| Spermatogonia | 67 (73.63%) | 71 (75.53%) | 90 (76.92%) | 68 (78.16%) | 69 (78.41%) | 71 (82.56%) |
| Spermatocytes | 24 (26.37%) | 23 (24.47%) | 27 (23.08%) | 19 (21.84%) | 19 (21.59%) | 15 (17.44%) |
