# Supplementary figures and images for: Testis transcriptome profiling identified genes involved in spermatogenic arrest of cattleyak
Source: PLoS One. 2020 Feb 24;15(2):e0229503. doi: 10.1371/journal.pone.0229503 (PMC7039509; doi:10.1371/journal.pone.0229503)

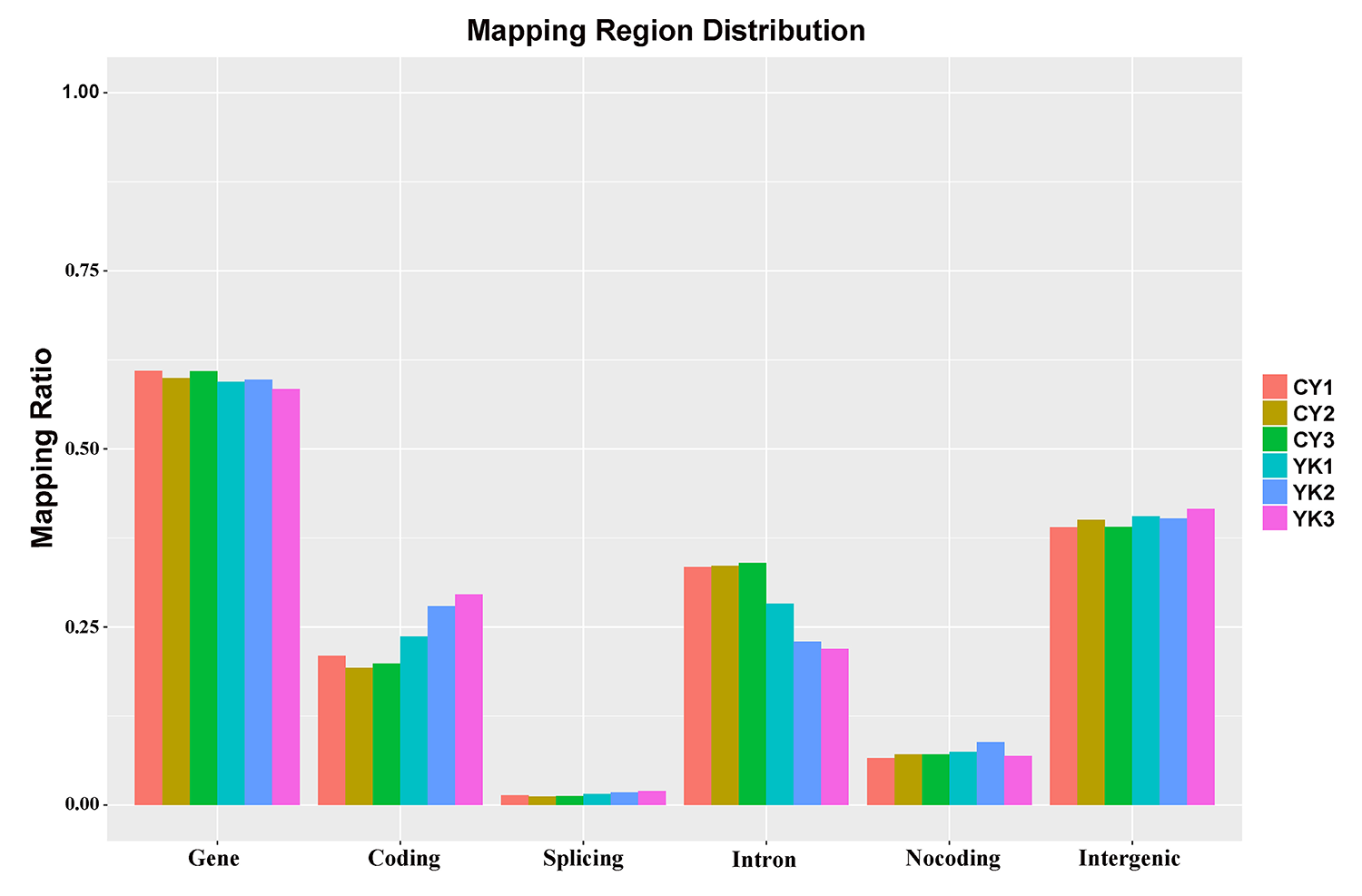

Supplement: S1 Fig — The ratio of reads from six samples mapped to the gene, coding region, splicing sites, introns and non-coding regions in the genome was indicated by different colors. The coding region includes exons and the exon coding sequence and the non-coding region includes 5' UTR, 3' UTR and non-coding RNA regions. (TIF) [file pone.0229503.s009.tif]

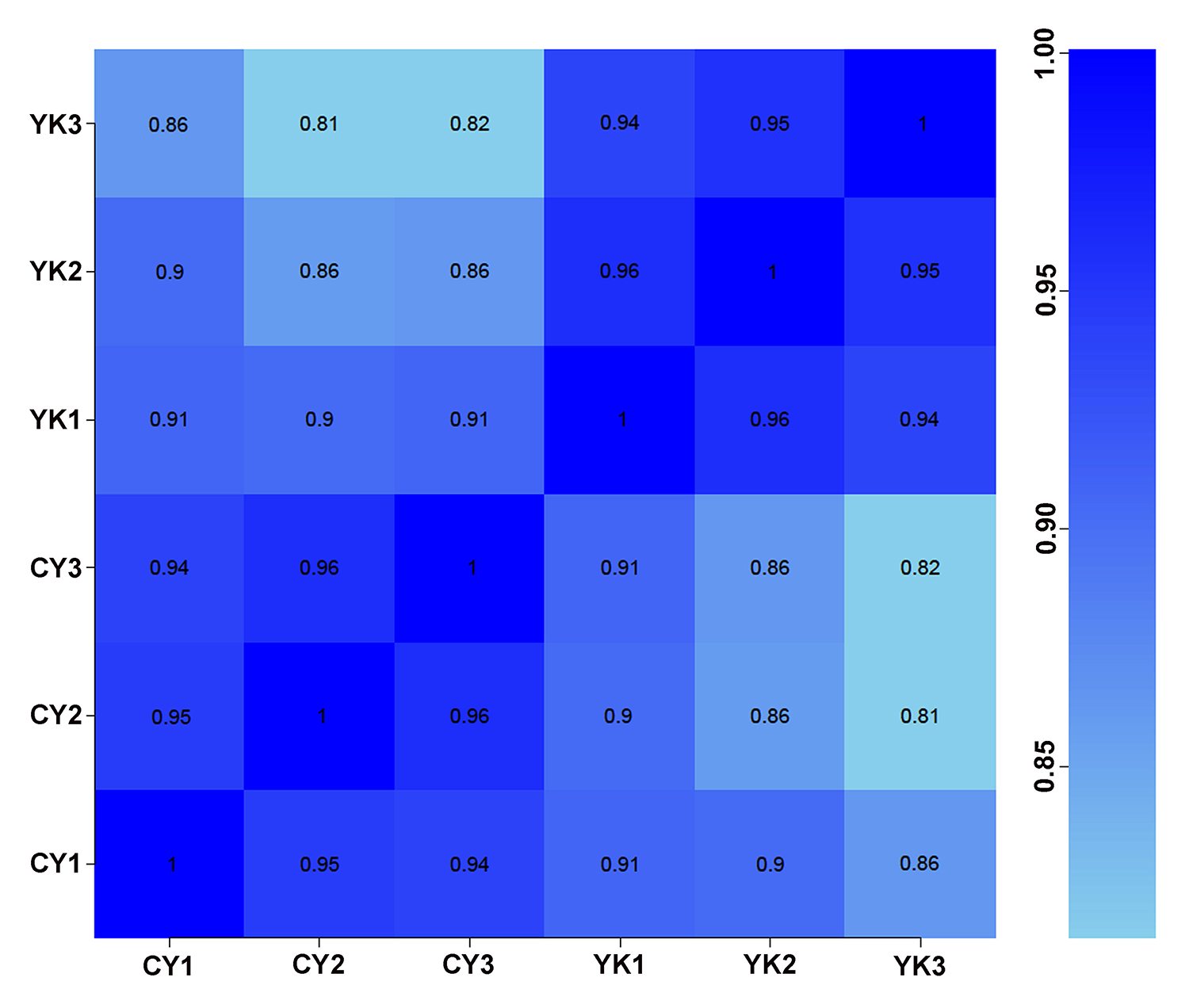

Supplement: S2 Fig — The darkness of the color is corresponding to the extent of correlations and the increase of Pearson correlation coefficients. (TIF) [file pone.0229503.s010.tif]

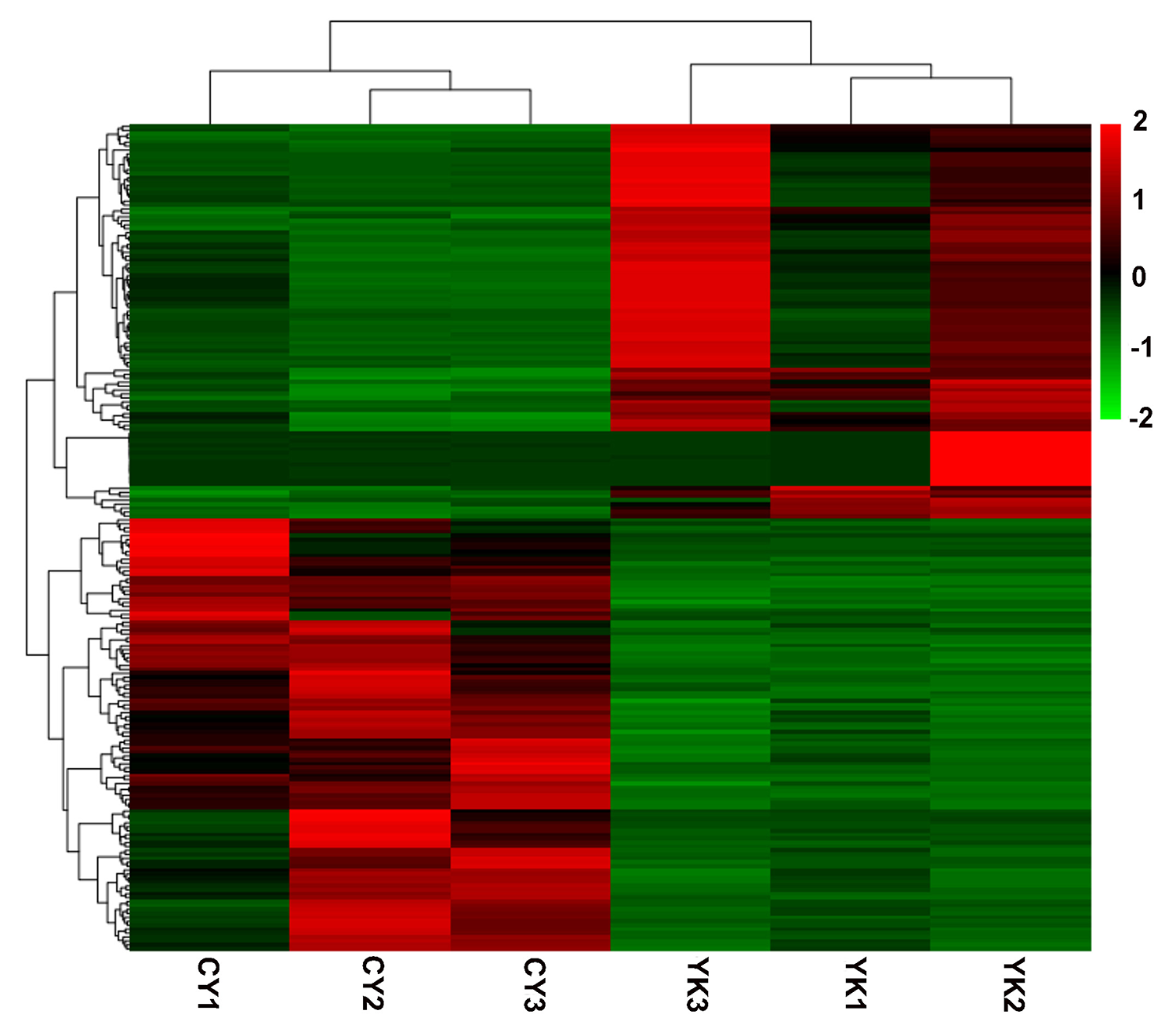

Supplement: S3 Fig — CY1 (Cattleyak 1), CY2 (Cattleyak 2), CY3 (Cattleyak 3), YK1 (Yak 1), YK2 (Yak 2), YK3 (Yak 3). (TIF) [file pone.0229503.s011.tif]

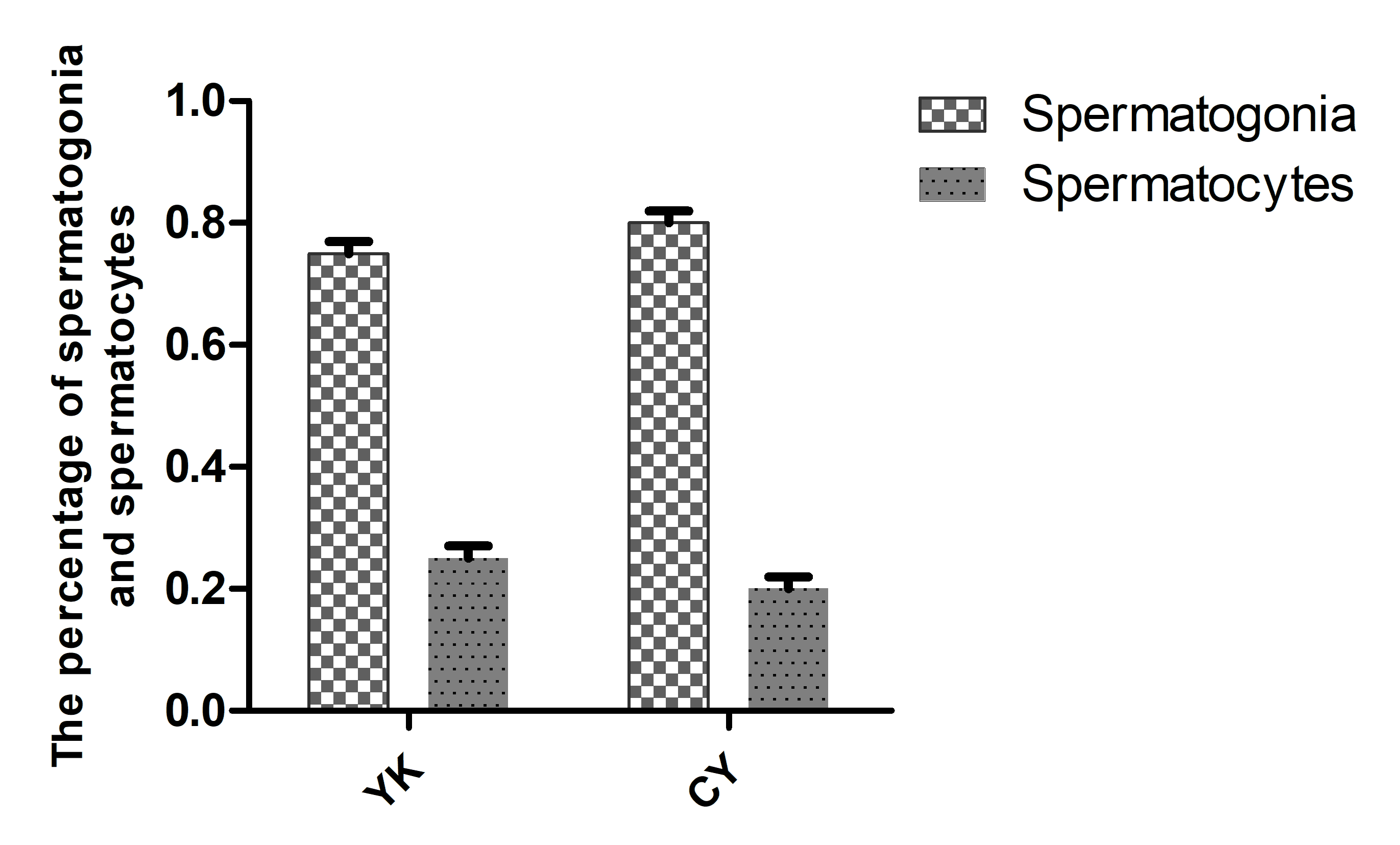

Supplement: S4 Fig — (TIF) [file pone.0229503.s012.tif]

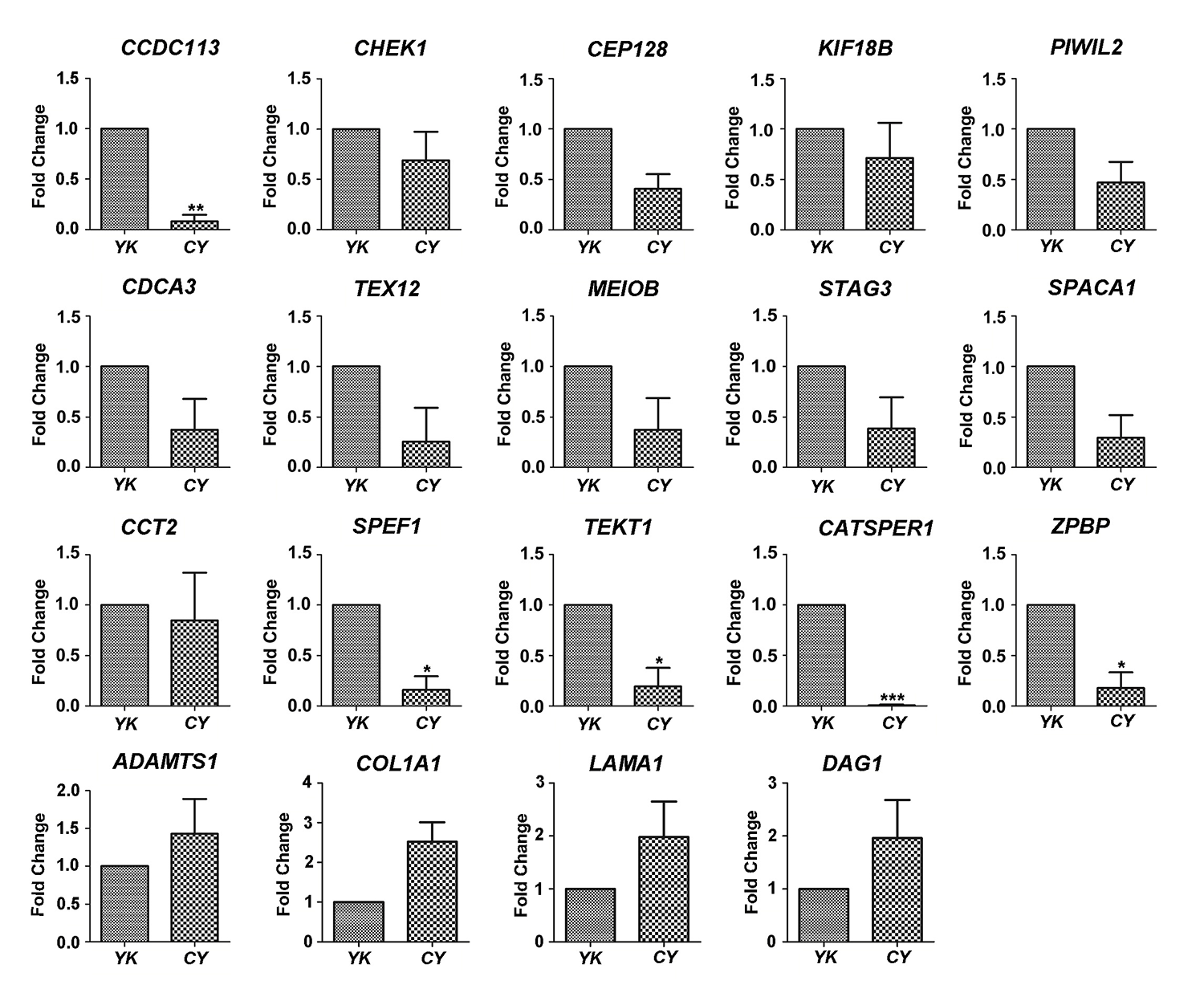

Supplement: S5 Fig — Data of qRT-PCR ware represented as mean±s.e.m. T-test was performed. Asterisks indicate statistical significance as compared to yak (*P < 0.05; **P < 0.01; ***P < 0.001). YK means yak, and CY means cattleyak. (TIF) [file pone.0229503.s013.tif]

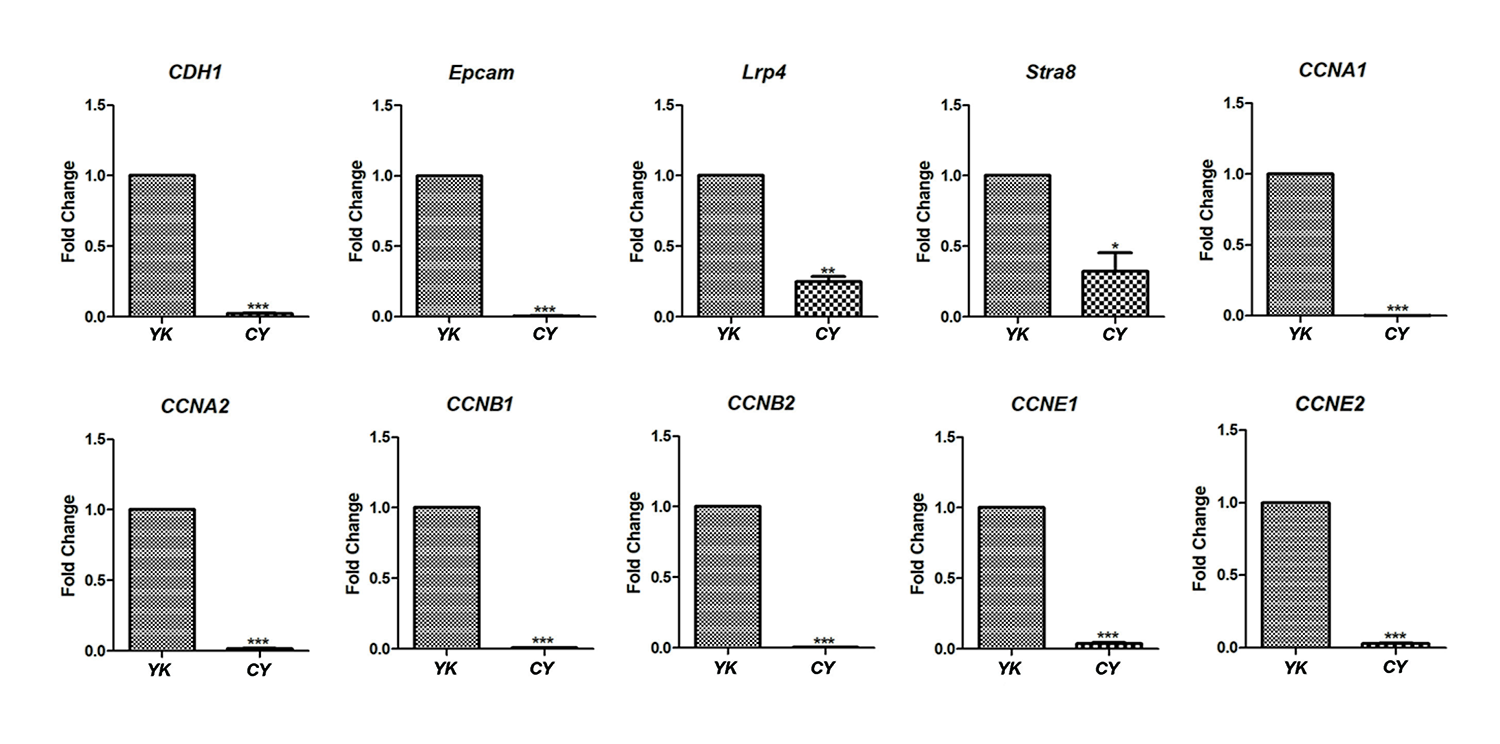

Supplement: S6 Fig — Asterisks indicate statistical significance as compared to yak (*P < 0.05; **P < 0.01; ***P < 0.001). (TIF) [file pone.0229503.s014.tif]
