# Supplementary material for: Testis transcriptome profiling identified genes involved in spermatogenic arrest of cattleyak
Source: PLoS One. 2020 Feb 24;15(2):e0229503. doi: 10.1371/journal.pone.0229503 (PMC7039509; doi:10.1371/journal.pone.0229503)

Full-length gels of Fig 5

Full-length gels of Fig 5 were shown below, and the sequence of all gels is *GAPDH*, *CD9*, *UCHL1*, *RET*, DL 2000 marker, *GAPDH*, *Tesmin*, *SYCP3* and *SYCP1*.

The original gel image for Figure 5E.

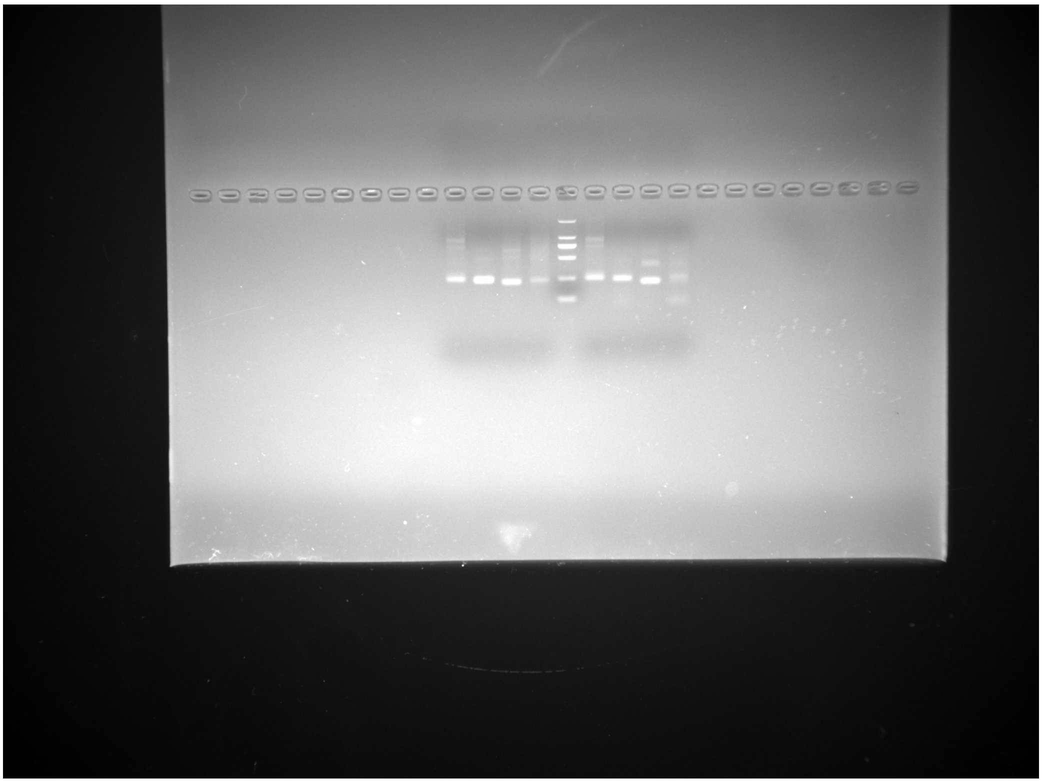

The original gel image for Figure 5F (inside the box).

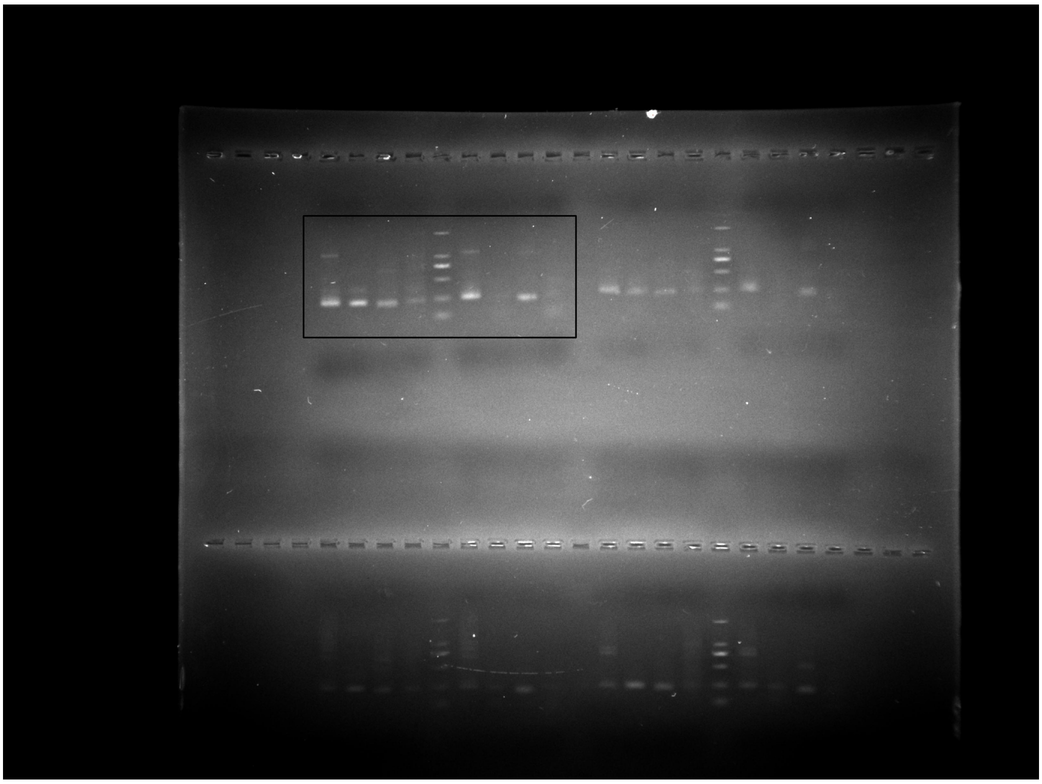

Supplement: S1 Raw Image — (PDF) [file pone.0229503.s015.pdf]
